# *Sargassum natans I* Algae: An Alternative for a Greener Approach for the Synthesis of ZnO Nanostructures with Biological and Environmental Applications

**DOI:** 10.3390/md21050297

**Published:** 2023-05-13

**Authors:** Jose Luis López-Miranda, Fabian Mares-Briones, Gustavo A. Molina, M. A. González-Reyna, Isaac Velázquez-Hernández, Beatriz Liliana España-Sánchez, Rodolfo Silva, Rodrigo Esparza, Miriam Estévez

**Affiliations:** 1 Centro de Física Aplicada y Tecnología Avanzada, Universidad Nacional Autónoma de México, Boulevard Juriquilla 3001, Querétaro 76230, Mexico; lopezfim@gmail.com (J.L.L.-M.); fabianmares@gmail.com (F.M.-B.); gamol@fata.unam.mx (G.A.M.); marlengonzalez@fata.unam.mx (M.A.G.-R.); isaacvh_qi@hotmail.com (I.V.-H.); resparza@fata.unam.mx (R.E.); 2 CONACYT_Centro de Investigación y Desarrollo Tecnológico en Electroquímica SC, Parque Tecnológico Querétaro s/n Sanfandila, Pedro Escobedo 76703, Mexico; lespana@cideteq.mx; 3 Instituto de Ingeniería, Universidad Nacional Autónoma de México, Edificio 17, Ciudad Universitaria, Coyoacán, Mexico City 04510, Mexico; rsilvac@iingen.unam.mx

**Keywords:** ZnO nanostructures, *Sargassum natans I*, green synthesis, antibacterial, photocatalytic, anti-inflammatory

## Abstract

In this work, the influence of the *Sargassum natans I* alga extract on the morphological characteristics of synthesized ZnO nanostructures, with potential biological and environmental applications, was evaluated. For this purpose, different ZnO geometries were synthesized by the co-precipitation method, using *Sargassum natans I* alga extract as stabilizing agent. Four extract volumes (5, 10, 20, and 50 mL) were evaluated to obtain the different nanostructures. Moreover, a sample by chemical synthesis, without the addition of extract, was prepared. The characterization of the ZnO samples was carried out by UV-Vis spectroscopy, FT-IR spectroscopy, X-ray diffraction, and scanning electron microscopy. The results showed that the *Sargassum* alga extract has a fundamental role in the stabilization process of the ZnO nanoparticles. In addition, it was shown that the increase in the *Sargassum* alga extract leads to preferential growth and arrangement, obtaining well-defined shaped particles. ZnO nanostructures demonstrated significant anti-inflammatory response by the in vitro egg albumin protein denaturation for biological purposes. Additionally, quantitative antibacterial analysis (AA) showed that the ZnO nanostructures synthesized with 10 and 20 mL of extract demonstrated high AA against Gram (+) *S. aureus* and moderate AA behavior against Gram (-) *P. aeruginosa*, depending on the ZnO arrangement induced by the *Sargassum natans I* alga extract and the nanoparticles’ concentration (ca. 3200 µg/mL). Additionally, ZnO samples were evaluated as photocatalytic materials through the degradation of organic dyes. Complete degradation of both methyl violet and malachite green were achieved using the ZnO sample synthesized with 50 mL of extract. In all cases, the well-defined morphology of ZnO induced by the *Sargassum natans I* alga extract played a key role in the combined biological/environmental performance.

## 1. Introduction

ZnO nanomaterials have aroused great interest due to their unique properties [1,2]. Some of the novel applications it has been given are photocatalytic [3], antibacterial [4], and biosensor [5] materials due to their low-cost synthesis and non-toxicity characteristics [6]. Many of its properties depend directly on size, morphology, and structure. In this sense, the synthesis of ZnO nanostructures with morphologies such as flowers [7], stars [8], wires [9], and rods [10] has been reported. Furthermore, some of these materials have exhibited an improvement in their properties, so that they can be used in different applications [11,12]. Remarkably, the biological behavior of ZnO nanostructures, such as anti-inflammatory and antibacterial, have gained attention due to the performance induced through the synergy between the size/morphology of nanoparticles and their increased specific surface area [13].

Additionally, the photocatalytic properties of ZnO are some of the most interesting due to their application in the degradation of organic dyes, which are highly used in the textile, cosmetic, and paper industries [14,15]. Unfortunately, many of these dyes have harmful effects on living beings [16]. At specific concentrations, they show a carcinogenic and mutagenic effect. Various methods have been used for its removal, including electrochemical [17], electroflotation [17], coagulation [18], and adsorption [15] methods. However, some are inefficient, and others are expensive due to the materials, equipment, and conditions necessary for pollutant removal. One of the most promising removal processes is photocatalysis, which involves using a photocatalyst material to carry out the dye degradation reaction. Different nanomaterials, such as Au [19], TiO_2_ [20], Fe_2_O_3_ [21], SnO_2_ [22], and ZnO [23], have been used for these purposes.

Other interesting properties that ZnO has shown are in biomedicine as antibacterial and anti-inflammatory materials. In the case of antibacterial activity, its behavior is associated with the generation of reactive oxygen species (ROS), ion diffusion, and the interaction of ZnO particles with cell membranes, resulting in the death of bacteria [24]. However, its use is only partially safe in some cases due to the precursors used. Various methods can be used to synthesize ZnO nanostructures, for example, solvothermal [25], sol-gel [26,27], reduction [28,29], microwave [30,31], and co-precipitation [32,33,34]. Compared with the different synthesis methods, the co-precipitation method is the most common synthesis for ZnO nanoparticles since it is easier, low-cost, environmentally friendly, capable of enormous-scale production, and employs precursors such as NaOH solutions, which allow for low-temperature processes. In the same way, the green synthesis of ZnO has also been reported [35,36,37]. Plant extracts such as *Pinus brutia* [38], *Citrus limon* [39], and *Ficus racemose* [40] have been used to synthesize different nanostructures of this oxide. These extracts provide the necessary compounds to carry out the precipitation reaction and, at the same time, act as stabilizing agents to control the morphology of the nanoparticles. This green method is safe, efficient, and environmentally friendly [37]. In the same way, it is possible to carry out the synthesis of nanomaterials using algae extracts [41]. Thus, the organic compounds from the alga coupled with the NaOH co-precipitation method can modify the surface chemistry of nanoparticles by changing their properties, controlling the size and morphology of the ZnO nanostructures [34]. In some regions of the world, certain algae have accelerated growth because of climate changes; this is the case of the *Sargassum* algae that reaches the Caribbean coasts [42].

Since 2014, a large amount of *Sargassum* algae has arrived in Mexico, increasing yearly [43]. This alga has generated various adverse effects, from environmental problems due to the alteration of the ecosystem to economic problems due to the decrease in tourism and substantial investment made by public and private entities to carry out the containment, collection, and management [43,44]. However, the problem continues beyond that point since the *Sargassum* algae, once collected, is taken to dumpsites, where it decomposes, leading to an important source of contamination. For this reason, various institutions and communities have tried to find a utility for this alga. Some products, such as blocks and shoe soles, have been made from *Sargassum* algae [42]. It has been reported that these *Sargassum* algae species have significant antioxidant activity, which can act as a reducing and stabilizing agent in synthesizing nanomaterials [45,46,47]. On the other hand, it has compounds that can be involved in the stabilization process, giving a particular morphology to the materials. In the case of the Mexican Caribbean, *Sargassum* algae comprises three different species: *fluitans III*, *natans VIII*, and *natans I* [48], the latter being the one with the highest proportion in some months of the year. In a previous work [45], the synthesis of ZnO nanoparticles using extracts from the mixture of these three species of *Sargassum* algae was reported. However, the results showed little control of the characteristics of the particles, which may be attributable to the difference in the chemical composition of the species involved. Therefore, the novelty of our work is to evaluate the stabilizing capacity of the *Sargassum natans I* alga extract through the synthesis of ZnO nanostructures, controlling the size and obtaining different morphologies. Additionally, we explore the multifunctional features of ZnO nanostructures, such as anti-inflammatory, antibacterial, and photocatalytic properties, aiming to produce eco-friendly nanomaterials with potential biological and environmental applications.

## 2. Results and Discussion

### 2.1. Physicochemical Characterization of ZnO Nanostructures Synthesized with Extracts of Sargassum natans I Alga

**Figure 1:**
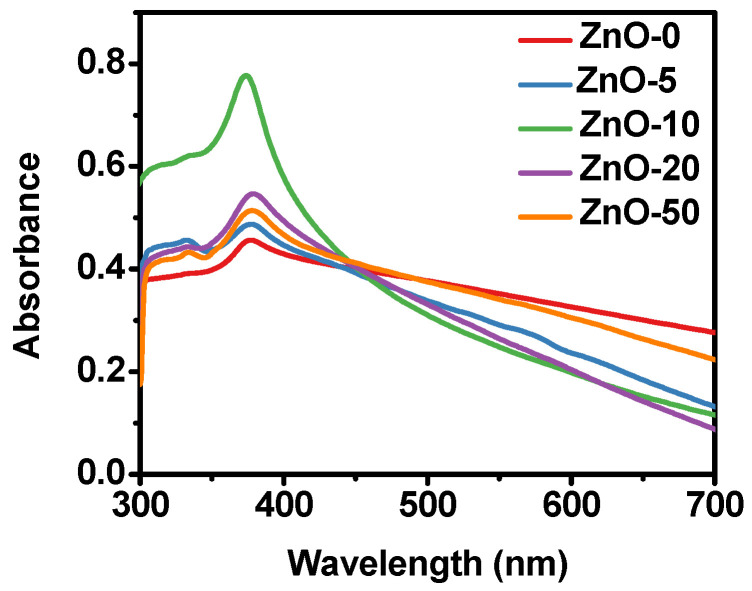
UV-Vis spectroscopy analysis of the ZnO synthesized without extract and ZnO nanostructures synthesized with different *Sargassum natans I* alga extract volumes.

UV-Vis spectroscopy is widely used for the identification of ZnO nanostructures. This material shows a characteristic band between 340 nm and 390 nm. Its position and width depend on the average particle size and size distribution [49]. The intensity of the band, as in other materials, is related to the concentration of particles present. Figure 1 shows the UV-Vis spectra of the ZnO nanostructures synthesized varying the volume of *Sargassum natans I* alga extract at 0, 5, 10, 20, and 50 mL; the samples obtained were named ZnO-0, ZnO-5, ZnO-10, ZnO-20, and ZnO-50, respectively As can be seen, when no extract was used, the ZnO band was at 378 nm and had a low intensity. Furthermore, the overall shape of this signal suggested a broad size distribution. When 5 mL of *Sargassum natans I* alga extract was used (ZnO-5), the band corresponding to zinc oxide was located at 377 nm, being of a higher intensity than the previous one. The spectrum corresponding to the sample ZnO-10 showed a very intense band centered at 373 nm, suggesting a high concentration of homogeneous nanoparticles. Moreover, this shift towards shorter wavelengths suggested a decrease in the average size of the nanoparticles. In the ZnO-20 and ZnO-50 spectra, an absorption band with similar characteristics was observed, a narrow band located at 376 nm, suggesting the presence of particles with similar physical characteristics. Due to the characteristics and position of the absorption bands, their sizes may be within the nanometric range, according to some reports. In addition, the influence of the extract amount on the concentration of nanoparticles was evident, which was associated with the absorption signal intensity. 

**Figure 2:**
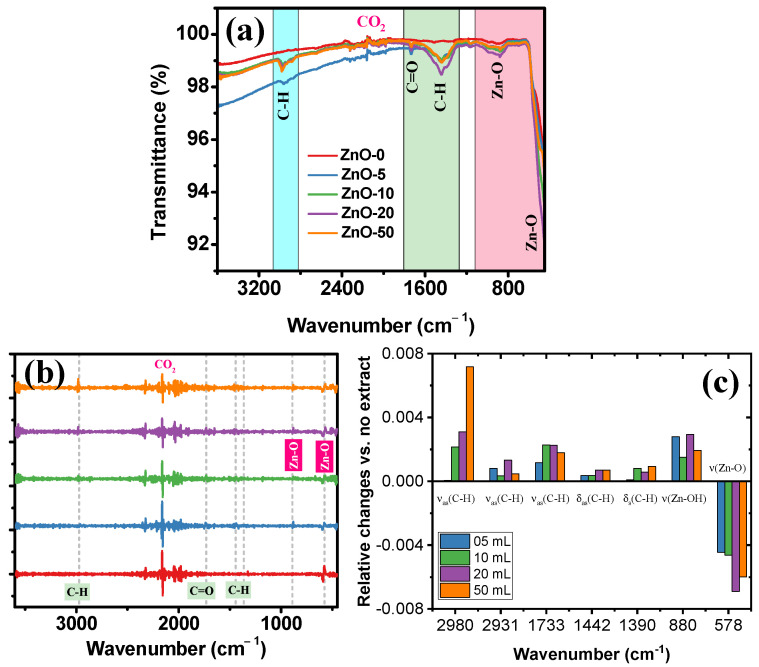
FTIR spectroscopy of the ZnO synthesized without extract and ZnO nanostructures synthesized with different *Sargassum natans I* alga extract volumes: (**a**) spectra, (**b**) second derivative analysis, and (**c**) relative changes in spectra signals.

The FTIR spectra obtained from the ZnO samples presented in Figure 2a show a signal centered at 881 cm^−1^, a small and wide band caused by the tetrahedral coordination of Zn, followed by a sharp and intense band centered around 575 cm^−1^, attributed to the stretching modes of ZnO [50,51]. An organic coating in the samples made with the *Sargassum natans I* alga extract caused some new bands at higher wavenumbers. First, at around 3000 cm^−1^, a band is presented caused by the symmetric and asymmetric stretch mode C-H bonds, followed by a band at around 1732 cm^−1^ describing simple carbonyl, while a methyl C-H asymmetric and symmetric bend produces the double band at 1470–1430/1380–1370 cm^−1^. As was expected, as the amount of extract was increased in the synthesis, and the bands related to stretch and bend modes of C-H bonds also increased. Then, a second derivative spectrum was calculated for each sample to identify small and nearby lying absorption peaks. The derivative process reduced the signal by approximately an order of magnitude and magnified noise by a factor of 6½ (e.g., the CO_2_ signal noise). It also required applying the Savizkye–Golay algorithm with ten smoothing points. As shown in Figure 2b, the bands associated with the organic part present a low signal-to-noise ratio because they had low intensities, contrary to the signal related to Zn-O, before the treatment. The relative changes caused by adding the extract to the synthesis in the second derivative peak heights by differentiating their value against the no-extract sample are presented in Figure 2c. First, the signals related to the organic part changed their intensities as the *Sargassum natans I* alga extract was added. Curiously, not all increased proportionally to this addition. Considering the possible fusion of some particles in the calcination process, the relative intensity changes suggested that the calcination could remove some functional groups, causing their disproportional increase.

**Figure 3:**
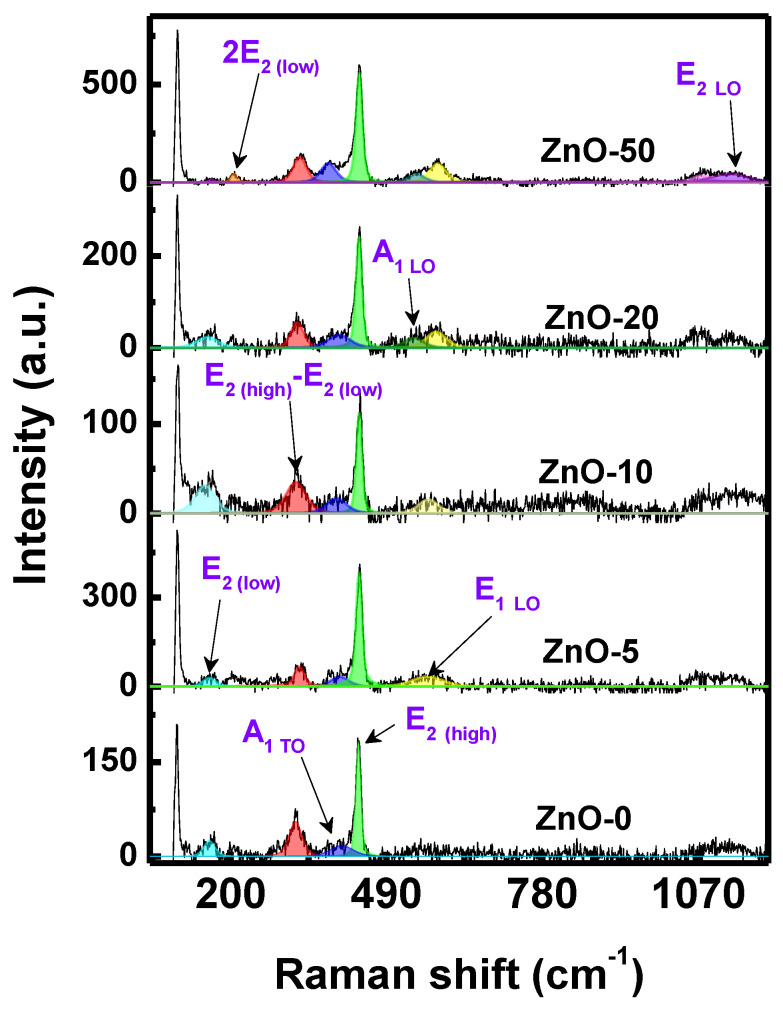
Raman spectra deconvoluted at λ 785 nm of the ZnO synthesized without extract and ZnO nanostructures synthesized with different *Sargassum natans I* alga extract volumes.

The Raman spectra of ZnO nanostructures synthesized with different concentrations of *Sargassum natans I* alga extract are shown in Figure 3. All samples presented a typical Raman spectrum related to a wurtzite-type structure of a ZnO [52]. Briefly, ZnO-50 presented the highest number of signals due to a possible formation of defects in the structure. These can be ascribable to the effect of temperature and the greater amount of *Sargassum natans I* alga extract used in the synthesis. In all the spectra, two signals were found around 160 and 438 cm^−1^, corresponding to the E_2 (low)_ and E_2 (high)_ modes, respectively [53]. Additionally, the E_2 (high)_ mode has been associated mainly with oxygen vibrations (green color marking) [52]. In several studies, a 438 cm^−1^ signal has been used to detect surface defects in the form of oxygen vacancies, where the E_2 (high)_ mode shifts to lower frequencies (lower energy) as oxygen vacancies increase [54]. Other vibrational modes were found: a second order signal (E_2 (high)_ − E_2 (low)_) around 330 cm^−1^, 382 cm^−1^ A_1 TO_, and the band envelope above 1000 cm^−1^ are attributed to harmonics and combined bands. The signal at 585 cm^−1^ was assigned to the E_1 LO_ mode, which was weak due to symmetry [55]. In addition, the signal at 547 cm^−1^ was identified as an additional signal arising from a second-order peak or due to oxygen vacancies. The samples synthesized with *Sargassum natans I* alga extract presented a greater oxygen deficiency than ZnO-0. It has been reported that there is a relationship between oxygen vacancies and photocatalytic activity; this relationship can be attributed to the fact that oxygen vacancy defects can induce new donor energy levels below their conduction band [53]. In addition, with a green method, defects in the form of oxygen vacancies were induced in the ZnO materials, which was practical since some techniques required harsh synthesis conditions or expensive facilities that limited their applications.

**Figure 4:**
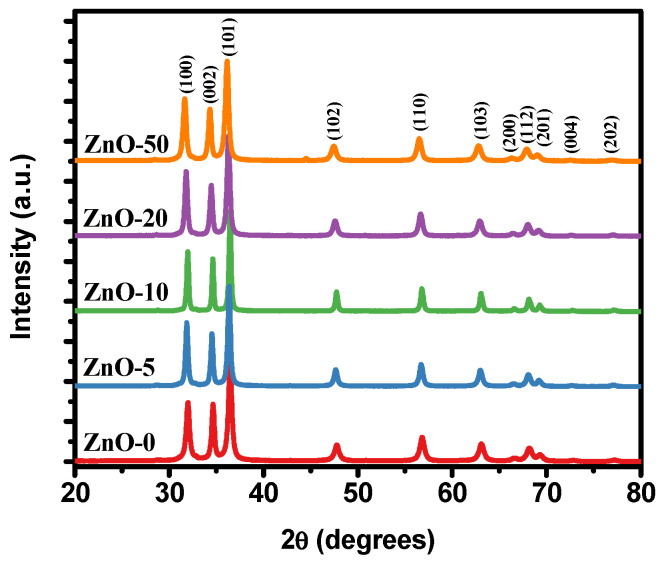
XRD patterns of the ZnO synthesized without extract and ZnO nanostructures synthesized with different *Sargassum natans I* alga extract volumes.

X-ray diffraction (XRD) analysis was carried out to determine the structural properties of the ZnO nanostructures. Figure 4 shows the XRD patterns of the ZnO particles synthesized with different extract volumes. All the XRD patterns show the characteristic peaks of the hexagonal wurtzite phase of the ZnO with space group P63mc [49]. The diffraction peaks at 2θ correspond to (100), (002), (101), (102), (110), (103), (200), (112), (201), (004), and (202) planes. The absence of diffraction peaks from other phases indicated the good crystallinity and high purity of the ZnO samples. The lattice parameters of the synthesized samples are summarized in Table 1. The parameters were calculated using the ReX program, which was based on the Rietveld method and allowed a quantitative and structural analysis of the X-ray diffraction data [56]. The lattice parameters of all the synthesized ZnO particles were similar to the ones reported in the JCPDS Card No. 36–1451, having a = 0.324982 nm and c = 0.520661 nm. However, there was a slight increase in the lattice parameters when the *Sargassum natans I* alga extract content increased too.

From the XRD patterns, the peak broadening indicated that small nanocrystals were present in the samples. The average crystallite size of ZnO samples was calculated according to the Debye–Scherrer equation [57]:

(1) $$D=\frac{K\lambda }{\beta cos\theta }$$

where *D* is the crystallite size (nm), *K* is a constant (0.9), *λ* is the wavelength of the X-ray radiation (Cu-Kα = 0.1541 nm), *β* is the full width at half maximum (FWHM) of the diffraction peak, and *θ* is the diffraction angle. In this case, the (101) plane was used because it is the most intense peak. The average crystallite size values are shown in Table 1. As observed, the crystallite size decreases with the increase in the *Sargassum natans I* alga extract content, from 36.31 nm to 20.94 nm for ZnO-0 and ZnO-50, respectively. This variation could be due to the change in size or shape of the ZnO nanostructures induced by the addition of the *Sargassum natans I* alga extract.

The relative intensity of the XRD patterns from the ZnO samples varied slightly with respect to that reported on the JCPDS Card No. 036-1451; therefore, a possible preferential orientation was obtained in the samples. The March–Dollase function (Equation (2)) represented the fraction of the preferred orientation:

(2) Pα=r2cos2α+1rsin2α−32

where *α* is the angle between the preferred orientation direction and the reciprocal-lattice vector direction for the Bragg peak that is corrected, and the March parameter *r* determines the preferred orientation; *r* = 1 indicates random orientation (no preferred orientation), *r* < 1 indicates a preferred orientation by plate-shaped crystals, and, finally, *r* > 1 indicates preferred orientation by needle-shaped crystals [58].

**Figure 5:**
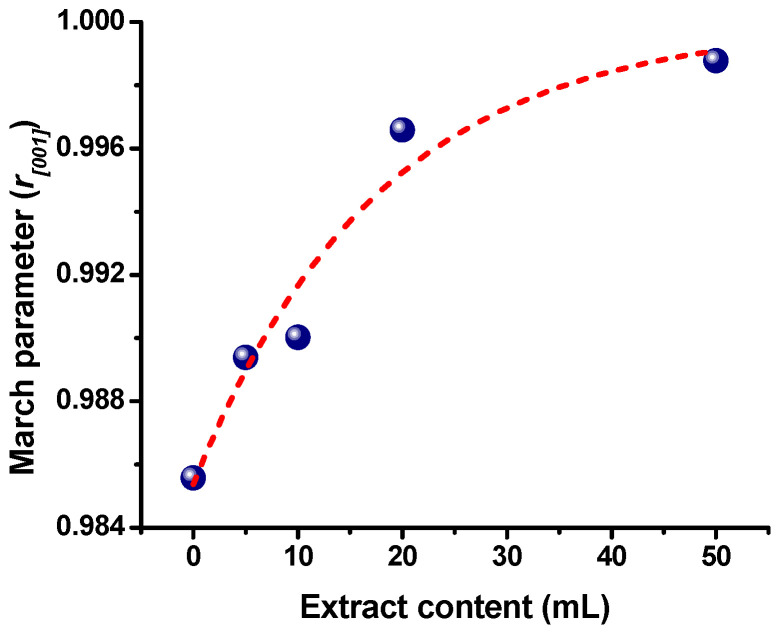
Variation in the parameter *r _[001]_* of the March–Dollase function with the *Sargassum natans I* alga extract content in the synthesized ZnO nanostructures.

Figure 5 exhibits the variation in the *Sargassum natans I* alga extract content used in synthesizing ZnO nanostructures with respect to the parameter r of the March–Dollase function. The (002) plane is the peak diffraction with a high difference in the relative intensity compared with the reported in the crystallographic card; therefore, the [001] orientation is selected for the analysis. As has been reported, the ZnO particles with flake shapes are (001) facets exposed; therefore, the relative intensity of the (002) plane increases in the XRD pattern [59]. From Figure 5, all the r values are less than one. However, the ZnO-0 sample has the lowest value, thus the flake-shaped is predominant. Nevertheless, with the increase in extract content in the synthesized ZnO nanostructures, the value of r tends to one, decreasing the flake shapes, and the crystals begin to have a random orientation (no preferred orientation). Preferential orientation can affect the particle shape and the physical and chemical properties of the ZnO particles [59].

**Figure 6:**
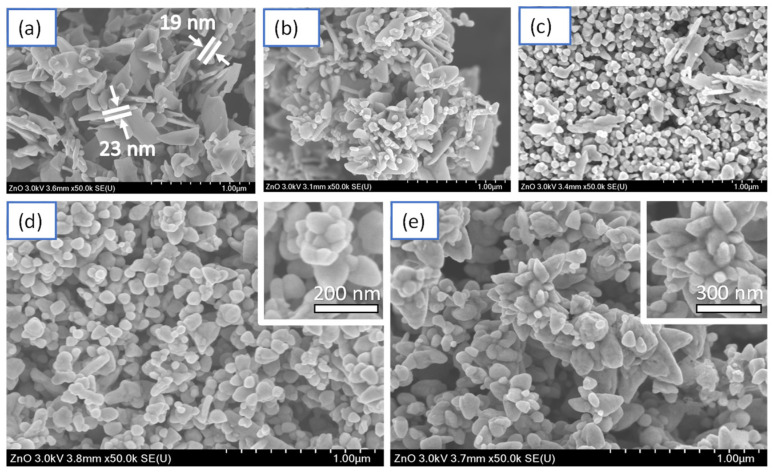
SEM analysis of the ZnO synthesized without extract and ZnO nanostructures synthesized with different *Sargassum natans I* alga extract volumes: (**a**) ZnO-0, (**b**) ZnO-5, (**c**) ZnO-10, (**d**) ZnO-20, and (**e**) ZnO-50.

The determination of the morphological characteristics, size, and distribution of the ZnO nanostructures was carried out by the SEM-SE (secondary electrons) technique. Figure 6a shows the micrograph corresponding to the ZnO-0 sample. A high density of laminar particles (flakes) with a low particle distribution can form large aggregates without coalescing. The individual length of the flakes only reaches maximum sizes of approximately 300 nm with an average thickness of 20 nm, classifying them as two-dimensional (2d) nanostructures. This behavior may be related to the absence of stabilizing agents that limit the growth of the particles along different preferential directions. According to the literature, the flake growth of ZnO occurs preferentially on the basal hexagonal close-packed (HCP) crystalline planes in the presence of shape-modifying agents. In addition, the relationship in the crystalline orientation in the ZnO plates is nonpolar in character. These results coincide perfectly with the studies obtained by the X-ray diffraction technique, showing a preferential orientation in the reflection peak (002) related to the formation of this type of two-dimensional structure; hence, the average particle sizes are acceptable for use in different applications.

In Figure 6b, the synthesized sample corresponds to the ZnO-5 sample, in which a significant morphological change is observed when compared with the sample obtained in the absence of the extract (Figure 6a). For these synthesis conditions, a bimodal morphological distribution is observed, giving rise to the presence of ZnO flakes and particles with a semispherical tendency; the dimensional conditions of the flakes remain constant. In contrast, the particles have average diameters of 50 nm. This behavior is attributed to the influence of the biomolecules in the *Sargassum* alga extract that act as a stabilizing agent, modifying the kinetics and partially limiting the growth of the particles. However, the sample indicates that the extract volume needs to be increased to stabilize all the products derived from the reaction. An inherent change in the morphology of the ZnO particles occurred with adding a larger volume of *Sargassum natans I* alga extract. Figure 6c corresponds to the synthesized sample using 10 mL of extract (sample ZnO-10). The micrograph shows a significant decrease in ZnO flakes and an increase in particles with semispherical morphology. This morphological change is synchronous with the reduction in the size of the particle and the dispersion of the sample, which presents an average diameter of 40 nm for semispherical particles. In different investigations, it has been reported that the decrease in particle size, as well as the morphological tendency, directly affects the antibacterial activity of nanomaterials because of the modification in the exposed surface area, being able to improve their activity and selectivity for a specific type of bacteria. This behavior confirms that the *Sargassum* alga extract plays an essential role in the stabilization and dispersion of the particles, controlling the size and morphology of ZnO.

Observing the micrograph corresponding to the ZnO-20 sample (Figure 6d), we can denote that the control in the morphology prevails with the increase in the *Sargassum natans I* alga extract volume, obtaining particles with a semispherical and subangular tendency with an average size of 60 nm. On the other hand, in the box shown in the micrograph, some particles tend to self-assemble on the facets, forming larger hierarchical structures made up of the two morphologies present in the sample. Finally, the analysis of the micrograph of the sample synthesized with the largest volume of *Sargassum natans I* alga extract (ZnO-50) is presented in Figure 6e. The image shows that by increasing the extract conditions during the synthesis reaction, the hemispherical morphology changes to subangular ZnO particles, which form hierarchical flower-like structures by self-assembly with sizes up to 0.5 µm. However, the tips of these particles are confined to the nanometer range, providing different possibilities for their use in various applications. Furthermore, this behavior occurs throughout the entire sample, indicating the participation of organic compounds from the *Sargassum natans I* alga extract, which plays a significant role in the stabilization, dispersion, and morphology of the sample.

Unlike the results reported by López-Miranda et al. [45], it should be noted that control over the size and morphology of the synthesized nanostructures was obtained in this case. Although it is true that the methodologies used were not comparable, the results obtained could be attributed to the control of the precipitation reaction, followed by the growth, aggregation, self-assembly, and, finally, a stabilization of the products, all this due to the influence of the organic compounds such as flavonoids and polyphenols from this *Sargassum* alga. According to the above, the reaction for the formation of the ZnO structures with different morphologies and sizes using the *Sargassum* alga extract could be established as follows [60,61,62]:

(3) $${Zn}^{2+}+2{OH}^{-}\overset{}{\to }Zn{\left(OH\right)}_{2}$$

(4) $$Zn{\left(OH\right)}_{2}+2{OH}^{-}\overset{}{\to }Zn{{\left(OH\right)}_{4}}^{2-}$$

(5) $$Zn{{\left(OH\right)}_{4}}^{2-}\overset{}{\to }ZnO+H_{2}O+{2OH}^{-}$$

In the ZnO co-precipitation reaction, NaOH acts as the precipitating agent. Zinc ions (Zn²⁺) and hydroxyl ions (OH⁻) combine to form zinc hydroxide (Zn(OH)₂), which precipitates as a white solid from the solution. At the same time, the *Sargassum natans I* alga extract acts as a stabilizing agent, controlling the size and morphology of the solids formed. Finally, ZnO results from the calcination of zinc hydroxide, giving rise to ZnO nanostructures with well-defined physical characteristics attributed to the *Sargassum natans I* alga extract.

Some of the properties exhibited by ZnO were associated with the band gap. It has been reported that this value is between 2.72 and 4.37 eV, depending on its size, morphology, and crystal size [63,64]. Figure S1 shows the band gap graphs obtained from the UV-Vis spectra using the Tauc equation [65]. The results show a high value (3.82 eV) for the sample in which the *Sargassum natans I* alga extract was not used. On the other hand, the ZnO samples synthesized with extract show a similar band gap between 3.24 and 3.44 eV. According to the SEM results, this value may be associated with the particle size since the lowest band corresponds to the ZnO-10 sample, which consists of nanoparticles between 40 and 60 nm. On the other hand, it has been reported that self-assembled structures can be highly active and, at the same time, exhibit low band gap values. For example, this is the case of the ZnO-50 sample made up of flower-like structures, whose band gap was 3.35 eV. These reduced band values imply that the electrons from the valence band can pass to the conduction band more easily; thus, these materials can be used in processes involving the transfer of electrons.

### 2.2. Anti-Inflammatory Activity of ZnO Nanostructures Synthesized with Extracts of Sargassum natans I Alga

Inflammation is a pathological condition that occurs after an injury or an infection, which restores the tissue microenvironment and cellular homeostasis. As the commercially available non-steroidal anti-inflammatory drugs (NSAID) have serious side effects that limit their usage for chronic inflammation diseases, ZnO nanostructures have attracted attention due to their excellent biomedical properties, such as enzyme regulation and protein synthesis. Additionally, ZnO nanoparticles are recognized as safe (GRAS) material by USA Food Drug and Administration (FDA).

**Figure 7:**
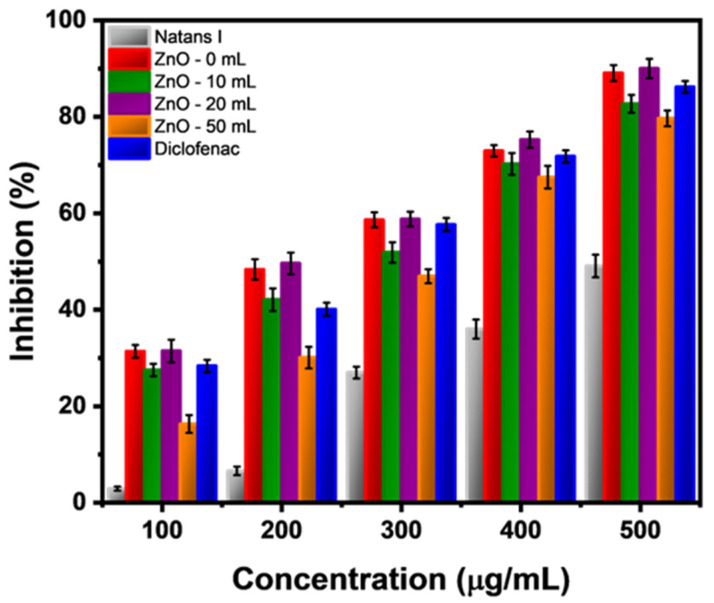
Comparison of the anti-inflammatory activity from all the ZnO nanostructures against DFS.

Due to the ZnO relevance, a protein denaturation (destruction of secondary and ternary protein structures) assay was performed to obtain its anti-inflammatory activity. This assay has been previously reported and is an attractive test to probe the ability to protect the biological function of the human body. The increment of absorbance in the sample against control (water) indicates higher protein stabilization (inhibition of denaturation) using the ZnO nanoparticles and a model drug such as DFS. The test results are observed in Figure 7 and Table 2, which are interesting to observe the effects of active metabolites that cap the nanoparticles synthesized by a green approach or the impact of the form obtained. The results demonstrate that the albumin denaturation inhibition increased as the sample concentration increased. The highest anti-inflammatory activity was observed when using the sample of ZnO-20 (90.01%), which was slightly higher than the chemically synthesized nanoparticles (ZnO-0) (89.07%) and the model drug (86.21%). The rest of the samples presented lower activity than the model drug, being 82.70% and 79.68% for ZnO-10 and ZnO-50, respectively. Additionally, it was observed that the lowest value obtained was when *Sargassum natans I* alga extract (49.09%) was used, proving that the stabilizing compounds in the ZnO surface influenced the anti-inflammatory properties. All these results were consistent with the rest of the concentrations used.

Since the nanostructures had diverse, active metabolites (in *Sargassum spp*.: carotenoids, phlorotannin, and some flavonoids), it was easier to have a higher inhibition effect. They might have presented covalent/non-covalent binding on the lysine, tryptophan, and tyrosine amino acids of the albumin ternary structure (loop 4, domain III), unlike ZnO nanostructures, which are first bonded to imidazole and then to the protein. It was expected to be a higher or similar activity than DFS and ZnO-0 in all the green synthesis samples, but, as observed, it was not. This behavior could be mainly attributed to a higher efficiency due to the surface area of active compounds and the shape/size of the nanostructures used.

As previously described in the SEM images, it is observed that there is a change in the size and morphology of ZnO nanostructures from 10 mL to 50 mL, severely affecting the anti-inflammatory activity. There was only one report on nanoparticles’ shapes/sizes and their heat transfer relations in solution, being a sample of gold nanoparticles, in which lower-shaped-like structures had higher local temperature increases than spherical-shaped nanoparticles at the same sizes and concentrations [66]. Thus, when the ZnO nanoparticles passed from a semispherical shape with width distribution to a spherical shape with a homogeneous distribution to a final flower-shaped form with also a homogenous distribution, there were changes in the local temperature from the ZnO nanoparticles to the protein, being from not a homogenous heat transfer to a highly increased heat transfer, due to the form and size causing higher denaturation of the albumin than expected.

Additionally, as observed in Table 2, to understand the effectiveness as an anti-inflammatory agent, the IC_50_ and the IC_90_ values were calculated for each sample, measuring the needed sample concentration to attain 50% and total anti-inflammatory activity. The lower these values, the higher the sample effectiveness since lower nanoparticle concentration is required to achieve the desired activity. It was observed that the IC_50_ and the IC_90_ values had the same tendencies described for the inhibition activity: ZnO 20 mL < ZnO 0 mL < DFS < ZnO 10 mL < ZnO 50 mL < *Natans I*. Additionally, the reported values in the present manuscript were in accordance with values reported in the literature using the same methodology [67,68].

### 2.3. Antibacterial Activity of ZnO Nanostructures Synthesized with Extracts of Sargassum natans I Alga

**Figure 8:**
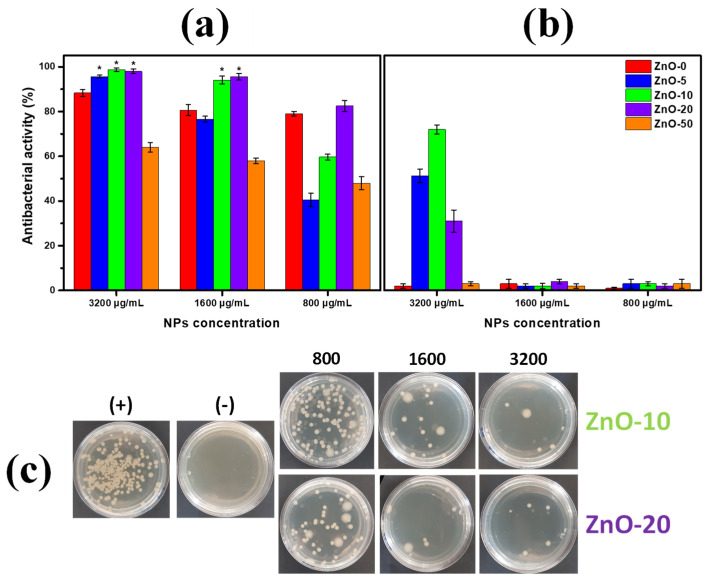
Antibacterial activity of ZnO nanostructures obtained from *Sargassum natans I* alga extracts by microdilution method after 3 h of contact against (**a**) *S. aureus* and (**b**) *P. aeruginosa*. (**c**) Representative photographs of *S. aureus* plates with different ZnO nanostructures (ZnO-10 and ZnO-20) under different nanoparticle amounts. * is considered significant, with a *p* value ≥ 0.05.

Over the last decade, the use of nanoparticles as antibacterial systems, such as silver or copper, has demonstrated relevance in avoiding the growth of pathogens, but their application is still under discussion due to their possible toxicity [69]. In this regard, ZnO has been recently positioned as a novel eco-friendly antimicrobial, with the advantage of its easy fabrication using natural sources. In addition, high-value-added ZnO nanostructures have been successfully explored using *Sargassum* alga waste. Antibacterial activity (AA) of obtained ZnO nanostructures was determined by the microdilution method after 3 h of contact. Figure 8a shows the AA of ZnO against *S. aureus*. Despite the moderate AA of ZnO obtained by the conventional chemical method (ZnO-0) of ca. 80%, the size/morphology of nanoparticles obtained by adding *Sargassum natans I* alga extract enhances the AA behavior. ZnO-10 shows an AA of ca. 99.03%at 3200 µg/mL and 93.82% at 1600 µg/mL. Additionally, ZnO-20 shows an AA of ca. 97.8% at 3200 µg/mL and 95.4% at 1600 µg/mL. The above indicates that the nanoparticles’ homogeneity and controlled size distribution of ZnO-10 induced by the *Sargassum* alga extract demonstrated by the UV-Vis results (Figure 1) positively impact the AA behavior. In addition, it is important to note that the lower band gap observed in Figure S1 in ZnO-10 allows the electron transference of Zn^2+^ species, reflected in the higher AA results. Otherwise, the poor AA in the ZnO-50 (of ca. 50–60%) suggests that the nanoparticles’ arrangement and agglomeration rests on the ion release, affecting the AA process [70]. Representative photographs of the AA of ZnO-10 and ZnO-20 against *S. aureus* are presented in Figure 8c.

The impact of the AA of ZnO nanostructures against Gram (-) *P. aeruginosa* is presented in Figure 8b. The above indicates that the differences in the bacterial cell wall composition between both microorganisms play a crucial role in the AA. Our results suggest that the double lipid layer of *P. aeruginosa* limits the ion transference of Zn^2+^ species and their interaction with the microorganism surface, reflected in the moderate AA of ZnO-10 of ca. 70% at 3200 µg/mL. Under this condition, the homogeneous nanoparticle size obtained from SEM micrographs (Figure 6c) of 40 nm promotes the interaction and penetration of the nanoparticles into the bacteria, inducing their growth inhibition [70]. In addition, as a control test, the extract was evaluated, showing no bactericidal properties (Figure S2), confirming that the antibacterial activity is attributed only to the ZnO nanostructures.

According to our results, the well-defined nanoparticles’ morphology induced by the controlled addition of *Sargassum natans I* alga extract promotes the fabrication of ZnO nanostructures with bactericidal characteristics. It has been claimed that the antibacterial mechanism of ZnO is mainly attributed to the electrostatic attraction of the Zn^2+^ species with the bacterial surface, producing severe membrane damage. This phenomenon allows the generation of reactive oxygen species (ROS), resulting in bacterial death at short contact time.

### 2.4. Photocatalytic Activity of ZnO Nanostructures Synthesized with Extracts of Sargassum natans I Alga

**Figure 9:**
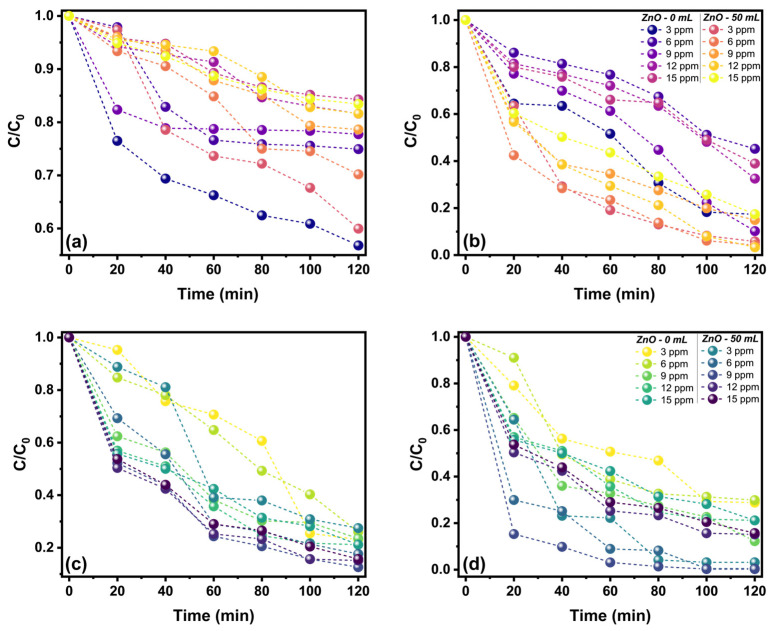
Effect of contact time on removing the dyes at different initial concentrations and radiation conditions: (**a**) MV on led, (**b**) MV on solar radiation, (**c**) MG on led, and (**d**) MG on solar radiation.

Figure 9 shows the effect of led or solar radiation on the methyl violet (MV) and malachite green (MG) dyes’ removal capacities. For both dyes, it is observed that increasing its initial concentration does not increase the dye removal efficiency. For the MV dye in led radiation (Figure 9a), it is observed that the ZnO-0 sample has the highest removal at an initial concentration of 6 μg/mL, while the ZnO-50 has the highest removal in solar radiation (Figure 9b) at an initial concentration of 12 μg/mL. Additionally, for this dye, it is observed that there is a significant increase in removal efficiency by changing from led to solar radiation, passing from 40% to total dye degradation. For the MG dye independent of the radiation, contrary to the effect observed in MV, almost total degradation can be achieved; enhancing with solar radiation produces a higher removal speed. It is observed that the ZnO -50 sample has the highest removal, independent of the radiation, being the highest at an initial concentration of 12 μg/mL in led radiation (Figure 9c), while in solar radiation at an initial concentration of 9 μg/mL (Figure 9d).

**Figure 10:**
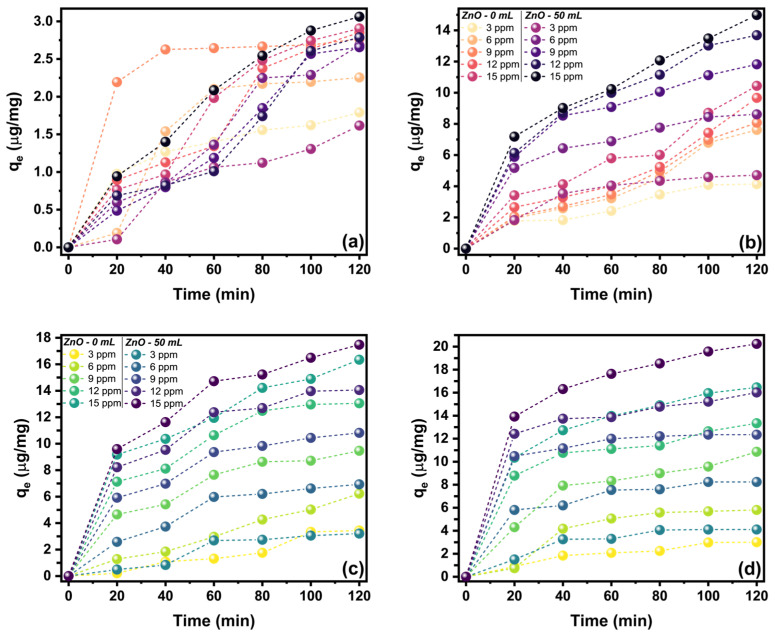
Effect of contact time on the adsorption capacity of ZnO nanostructures at different initial dye concentrations and radiation conditions: (**a**) MV on led, (**b**) MV on solar radiation, (**c**) MG on led, and (**d**) MG on solar radiation.

The effect of the contact time of the ZnO nanostructures on the adsorption capacity of MV and MG can be observed in Figure 10. It is observed that different times to reach the adsorption equilibrium are needed depending on the dye, radiation condition, and type of synthesis. For the MV adsorption using ZnO-0 on led radiation (Figure 10a), it is observed that equilibrium can be achieved at 80 min, and, on solar radiation (Figure 10b), it requires more than 100 min. In both cases, the maximum adsorption capacity (*q_m_*) is achieved at the 15 μg/mL being of 2.91 and 10.44 μg/mg. For the same dye (MV), but when using the ZnO-50 sample for both types of radiation, it is observed that more than 100 min is required to achieve *q_m_*, and the highest values can be observed at 3.06 and 14.99 μg/mg, as can be observed in Figure 10a,b, respectively. MG dye observed in Figure 10c shows that the equilibrium can be achieved approximately at 80 min for both types of ZnO nanostructures in led radiation, and the value of *q_m_* for ZnO-0 is 16.34 μg/mg and for ZnO-50 is 17.48 μg/mg, at 15 μg/mL dye equilibrium concentration. A similar case is shown in Figure 10d for both ZnO nanostructures since equilibrium is achieved at 80 min, and values for *q_m_* at dye equilibrium concentration are 16.46 μg/mg and 20.24 μg/mg for ZnO-0 and ZnO-50, respectively.

**Figure 11:**
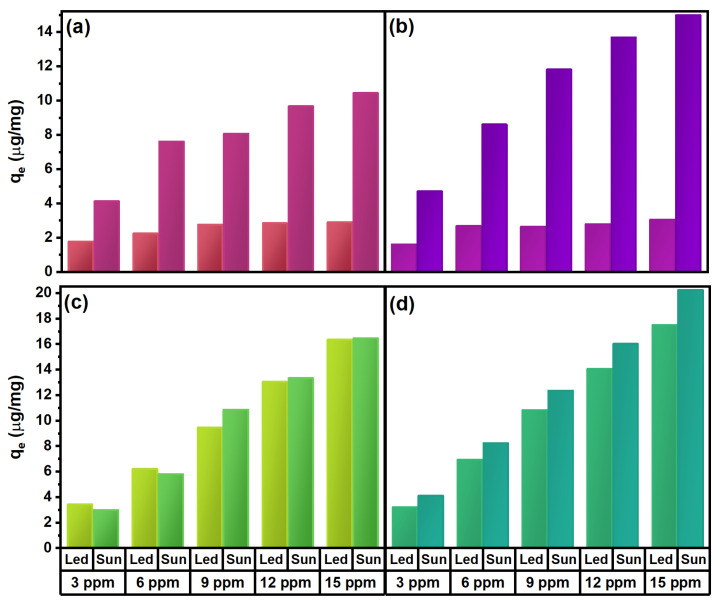
Comparison of maximum adsorption capacity of ZnO nanoparticles at an equilibrium concentration of dye solution at different radiation conditions: (**a**) MV onto ZnO-0, (**b**) MV onto ZnO, (**c**) MG onto ZnO-0 and (**d**) MG onto ZnO-50.

The differences between the ZnO samples using the different types of radiation can be observed in Figure 11a,b. As observed and previously described, adsorption is most effective at the highest dye equilibrium concentration, using the green synthesized ZnO-50 nanostructures, and clearly when using solar radiation. When using MG dye, the effect of solar radiation is not as effective as in the case of MV. As shown in Figure 11c,d, the impact of using different radiation types is ineligible in *q_m_* for the ZnO-0 sample and the ZnO-50 samples; the effect is slightly increased, by an average of 20%, for all the concentrations used, proving the effectiveness of the ZnO nanostructures for the adsorption of this dye without catalytic effects.

To further analyze the adsorption kinetics for the removal of MV and MG using the proposed ZnO nanostructures, three of the most used kinetic models, namely, Lagergren pseudo-first-order (PFO, Equation (6)), Ho and Mckay pseudo-second-order (PSO, Equation (7)), and the intraparticle diffusion model (IPD, Equation (8)), were applied to the obtained data. The equations are presented in order as follows.

(6) $$\ln\left(q_{e}-q_{t}\right)=\ln\left(q_{e}\right)-k_{1}t$$

(7) $$\frac{t}{q_{t}}=\frac{1}{k_{2}q_{e}^{2}}+\frac{t}{q_{e}}$$

(8) $$q_{t}=k_{i}\sqrt{t}+C_{i}$$

where *q_e_* (μg/mg) and *q_t_* (μg/mg) are the adsorption capacity at the equilibrium and at a given time *t*; *k*_1_ (mg/μg·min), *k*_2_ (mg/μg·min), and *k_i_* (μg/g·min^0.5^) are the constants of the PFO, PSO, and IPD models, respectively; and *C_i_* (μg/mg) is the intercept of the IPD model. The kinetic parameters obtained after the fitted plots of the adsorption kinetics models have been summarized in Table 3. Each proposed model’s viability was checked by its corresponding correlation coefficient (*R*^2^). For MV dye, it was observed that the *R*^2^ values for the Lagergren model were the biggest in both radiation conditions. It suggested that the pseudo-first-order equation was best to predict its kinetics; with this, it could be concluded that the adsorption rate was proportional to the difference between the adsorption capacity at equilibrium and determinate given time for MV, being said differently, the adsorption of MV on ZnO nanoparticles was controlled by mass transfer. Conversely, in both radiation conditions for the ZnO nanostructures tested for MG dye, the Ho and Mckay model had the highest correlation values, suggesting that the pseudo-second-order equation was the best to predict its kinetics. For MG dye, it could be concluded that its adsorption was being controlled by a chemical reaction progress involving electron sharing/transfer.

In all cases, the IPD model presented linearity in all the samples and adjusted over a correlation coefficient of *R*^2^ > 0.9, indicating that the adsorption on the external surface of the ZnO nanoparticles was slow. Additionally, from the values obtained for the intercept *C_i_* for all the conditions tested were non-zero. Usually, this value was related to the thickness of the boundary layer, and the larger it was the more significant the effect of the boundary layer. Negative intercepts indicated that surface reaction control was slowing the intraparticle diffusion (ZnO-0 and ZnO-50 in led light for MV). These results revealed that the adsorption process was not controlled only by intraparticle diffusion but involved a complex mechanism where both chemical and physical adsorption were included.

Additionally, the adsorption process could be further analyzed using the term defined as the initial adsorption factor (*R_i_*) of the intraparticle diffusion model, which could be represented in terms of the ratio of the initial adsorption amount to the final adsorption amount:

(9) $$R_{i}=1-\left(\frac{C_{i}}{q_{e}}\right)$$

The diffusion process could be characterized by different initial adsorption behavior depending on the value obtained for the *R_i_* values. Three different behaviors were presented depending on the ZnO nanoparticles, the dye, and the radiation. First, being under the zone 0 regiment, in which there was no initial adsorption, were the ZnO-0 samples in both radiation conditions and ZnO-50 in led for MV. Next were three other samples under the zone 1 regiment, in which weak initial adsorption took place: the last MV ZnO-50 sample in solar radiation and both ZnO-0 and ZnO-50 samples under led radiation for MG dye. The remaining two samples, ZnO-0 and ZnO-50 under solar radiation, were cataloged in the zone 2 regiment, in which intermediately initial adsorption was presented. These results reinforced the idea that surface adsorption was not the dominant process in dye degradation.

The relationship between the adsorption capacity and the equilibrium concentration in the solution of the selected dyes at the different radiation conditions was already observed in Figure 11, where the adsorption capacity increased with the increasing of the equilibrium concentration until the adsorption saturation was achieved. The effect could be attributed to the fact that a higher driving force was provided at a higher initial dye concentration, and the diffusion of the dye molecules on the surfaces of the ZnO nanostructures would be accelerated. Two-adsorption isotherm models were tested with the Langmuir (Equation (10)) and the Freundlich equation (Equation (11)) to further describe the adsorption at equilibrium:

(10) $$\frac{C_{e}}{q_{e}}=\frac{1}{k_{L}q_{m}}+\frac{C_{e}}{q_{m}}$$

(11) $$\log\left(q_{e}\right)=\frac{1}{n}\log\left(C_{e}\right)+\log\left(k_{F}\right)$$

where $C_{e}$ (μg/mL) is the equilibrium concentration of the dye solution, $q_{m}$ (μg/mg) is the maximum adsorption capacity, $k_{L}$ (mL/μg) is the equilibrium adsorption constant for the Langmuir isotherm model, $k_{F}$ (mL/μg) is the Freundlich constant associated with the adsorption driving force, and 1/*n* is the non-linearity between solution concentration and adsorption.

The calculated parameters and correlation coefficients for the adsorption of MV and MG dye onto the ZnO nanostructures are summarized in Table 4. Depending on the dye used for adsorption, independently of the radiation source, is the correlation value. For MV using both ZnO samples, higher correlation values were obtained using the Langmuir model, which described that the monolayer adsorption happened on a homogeneous adsorbent surface. The adsorption capacity was calculated as 3.52 and 16 μg/mL for led and solar radiation on ZnO-0, respectively, and 3.68 and 33.11 μg/mL for led and solar radiation on ZnO-50, respectively. MG using both ZnO nanostructures had higher correlation values with the Freundlich model, indicating heterogeneous surface adsorption and multilayer adsorption under various non-ideal conditions. The 1/n > 1 suggested the preference for a chemical process of adsorption.

## 3. Materials and Methods

### 3.1. Materials

*Sargassum* was collected in Puerto Morelos, Quintana Roo (20°50′44.1″ N, 86°52′35.5″ W) and classified according to the different species present (Figure S3). The *Sargassum natans I* alga was washed to remove sand and was dried for 24 h using a solar desiccator that operated between 40 and 45 °C; then, it was stored in the shade for future use. The precursor salts were zinc acetate dihydrate (99.99% trace metals basis) and NaOH reagent grade. The organic dyes were methyl violet and malachite green, both reagent grade. All reagents were purchased from Sigma Aldrich. Aqueous solutions and dilutions were prepared using deionized water.

### 3.2. Preparation of Sargassum natans I Alga Extract

The *Sargassum natans I* alga was washed several times with water to remove sand and other impurities and dried at room temperature. Next, the alga extract was prepared by mixing 1 g of *Sargassum natans I* alga with 50 mL of deionized water. This mixture was heated at 60 °C for 30 min under magnetic stirring. Subsequently, it was passed through filter paper, and the liquid phase was stored under refrigeration. A typical image of the extract is shown in Figure S4.

### 3.3. Synthesis of ZnO Nanostructures Using Sargassum natans I Alga Extract

The synthesis of ZnO nanostructures was carried out by a method consisting of two steps. The first step involved mixing 20 mL of an aqueous zinc acetate solution (50 mM) with 10 mL of 10 mM NaOH and *Sargassum natans I* alga extract at 80 °C under magnetic stirring for 2 h. The extract volume varied at 0, 5, 10, 20, and 50 mL, and the samples obtained were named ZnO-0, ZnO-5, ZnO-10, ZnO-20, and ZnO-50, respectively. After letting the mixture settle for 24 h, the supernatant of the sample was removed, and washing for three cycles was carried out, which consisted of adding deionized water, redispersing the sample in an ultrasonic bath, and centrifuging to precipitate the ZnO. Next, the sample was dried in a furnace at 60 °C. The second step involved subjecting the sample to a thermal treatment, heating at 600 °C for 4 h. Finally, the sample was collected and stored for later use and characterization.

### 3.4. Characterization

Different characterization techniques evaluated the physicochemical characteristics of ZnO nanostructures. The presence of ZnO was determined by UV-Vis spectroscopy, for which aqueous solutions were prepared at a concentration of 0.1 mg/mL. The analysis was carried out in a Metash 6000M spectrophotometer employing quartz cells. The scan was performed from 200 nm to 600 nm with a step of 1 nm. To ensure that the samples were under the same conditions, they were kept dispersed under an ultrasonic bath until the analysis was performed. The chemical bonds associated with ZnO and the extract were determined by FT-IR spectroscopy using Perkin Elmer, Spectrum Two equipment. The analysis was carried out in a wavelength range of 400 cm^−1^ to 3000 cm^−1^. Micro-Raman spectroscopy was used to determine crystal symmetry and local structural changes. A HORIBA XploRa spectroscope (Kyoto, Japan), equipped with an asymmetric crossed Czerny–Turner spectrometer, was used. The spectral range for all samples was from 50 to 1300 cm^−1^, and the excitation time was 10 s in 4 cycles. The structural evaluation and the phases present in the samples were determined by X-ray diffraction employing a Rigaku Ultima IV diffractometer. The particle size was evaluated by dynamic light scattering (DLS), placing the liquid samples in disposable cells, carrying out the study in a Litesizer 500 Anton Paar equipment. Finally, the sizes and morphologies of the ZnO samples were analyzed with a Hitachi SU8230 cold-field emission scanning electron microscope.

### 3.5. Antibacterial Activity

Quantitative assays of antibacterial activity (AA) of ZnO by microdilution method were performed using strains of Gram (+) *S. aureus* # 6538 and Gram (−) *P. aeruginosa* #13388 growth in Mueller Hinton media (BD-Bioxon). An inoculum of 16 h of growth of each microorganism was adjusted to the final concentration of 2 × 10^5^ CFU/mL (working inoculum) by optical density at 600 nm. ZnO nanostructures were suspended in sterile phosphate buffer (PBS, pH 7.2) with Tween 80 1% *v*/*v* as NPs dispersant. Different ZnO concentrations (3200, 1600, and 800 µg/mL) were evaluated through the interaction of an equal volume of NPs/ bacteria in a 96-well plate and incubated for 3 h at 37 °C, considering the mix 1:1 of the working inoculum with PBS/Tween without NPs as positive control (+) and the PBS/Tween with MH media as negative control (−). Three independent experiments were performed by duplicates. After ZnO/bacteria interaction, an aliquot of 50 µL was plated in MH plates and incubated for 16 h at 37 °C. Survival bacterial recovery (AA) was calculated according to our previous works [45,47].

### 3.6. Study of Anti-Inflammatory Properties

The in vitro anti-inflammatory activity of the ZnO nanostructures was measured using the proposed methodology from Chandra et al. Initially, a 2 mL of the tested ZnO-α mL nanoparticles (α = 0, 10, 20, and 50) solution at different concentrations was prepared. The concentrations of the samples tested were 100, 200, 300, 400, and 500 μg/mL. Then, 0.2 mL of egg albumin from a fresh egg was added to the samples and stoppered at 5 mL using a phosphate buffer of pH = 6.4. The homogenized solutions were incubated at 37 ± 2 °C using a convection oven (DKN302, Yamato Scientific Co., Ltd.; Tokyo, Japan) for fifteen minutes. After the elapsed time, the chamber temperature increased to 70 °C, and the reaction was left for five additional minutes. Finally, the samples were left to stand at room temperature and cooling; the solution was measured using UV-Vis spectroscopy at a wavelength of 660 nm. All the analyses were performed in triplicate, and the percentage of inhibition of protein denaturation was calculated as follows:

(12) $$nhibition\left(\%\right)=\left(1-\frac{A_{S}}{A_{C}}\right)\times 100$$

where *A_S_* is the absorbance of the sample, and *A_C_* is the control absorbance. The control consisted of an additional test using 2 mL of deionized water as a sample. Additionally, sodium diclofenac salt (DFS) was measured at the same concentration of the samples to be used as a drug reference for the test.

### 3.7. Evaluation of Photocatalytic Activity

The methyl violet (MV) or malachite green (MG) dyes were prepared using deionized water in a 1 L volumetric flask. The kinetic tests were performed by mixing 1 mL of ZnO nanostructures samples at an 8 mg/mL concentration and 10 mL of the dye at a determined concentration at room temperature. The effect of different light sources was studied by letting the ZnO+ dye solution stand using a 45 W led light panel at room temperature (Led) and using the midday solar light (UV index = 11, Solar) at ambient temperature. The adsorption isotherm studies were carried out using the different initial concentrations of the dye from 3 μg/mL up to 15 μg/mL. The samples evaluated were ZnO-0 and ZnO-50, which were selected to evaluate the behavior of the sample obtained with the highest volume of *Sargassum natans I* alga extract and compare the results with the synthesized sample without extract.

The ZnO+ dye solution was monitored at different time intervals (0–120 min), where the supernatant liquid (approximately 2.5 mL) was sampled using a syringe; the remaining solids were removed using a 0.22 μm membrane filter. Then, the dye concentration was determined at different wavelengths (MV@589 nm and MG@617 nm) using a Lambda 365 UV-Vis spectrometer (Perkin Elmer, Waltham, MA, USA). The dye fractional dye removal was calculated using the ratio, while the adsorption capacity was calculated using the difference between the dye concentration in the aqueous solutions before and after the and the adsorption capacity:

(13) $$r=\frac{C_{t}}{C_{0}}$$

(14) $$q_{e}=\frac{V}{w}\left(C_{0}-C_{t}\right)$$

where *r* is the dimensionless fractional removal, *q_e_* is the equilibrium concentration of the dye on the adsorbent (μg/mg), *C*_0_ is the initial concentration of the dye solution, *C_t_* is the dye concentration at a given time *t*, *V* is the volume of dye solution (mL), and *w* is the weight of ZnO (mg) nanostructures used. All experiments were duplicated; only the average results are reported in the present manuscript.

## 4. Conclusions

In the present work, different multifunctional ZnO nanostructures were synthesized using the seaweed *Sargassum natans I* alga extract. Furthermore, the influence of the extract volume as stabilizing agent was evaluated at different conditions (0, 5, 10, 20, and 50 mL) for the formation of ZnO nanostructures. ZnO was obtained by calcinating zinc hydroxide, the product of the co-precipitation reaction between zinc acetate and NaOH. The *Sargassum* alga extract showed stabilizing properties with the ability to maintain the particles at the nanometer scale or to promote the self-assembly of specific morphologies, depending on the volume used. Furthermore, the microstructural characteristics of the synthesized samples were determined by the characterization techniques: UV-Vis, FTIR, RAMAN, XRD, and SEM.

Using UV-Vis, the formation of ZnO particles was determined due to the presence of characteristic absorption bands in the spectrum located between 370 and 380 nm, indicating the existence of particles in suspension. Furthermore, FT-IR analysis showed signals associated with Zn tetrahedral coordination and ZnO stretching modes. According to the XRD results obtained, all the samples were composed of a single phase corresponding to the hexagonal crystalline structure of wurtzite, confirming the formation of high-purity ZnO. On the other hand, it was determined that there was a relationship between the decrease in crystallite size and a growth control referring to preferential orientations (002), depending on the increase in the extract. These results were corroborated through the SEM technique, confirming the influence of the *Sargassum natans I* alga extract on the stabilization of ZnO, obtaining particles with flake-like morphologies for the ZnO-0 sample. In contrast, semispherical nanoparticles were obtained with increased extract (ZnO-5 and ZnO-10). However, a self-assembly phenomenon occurred for greater volumes of extract (ZnO-20 and ZnO-50), forming structures with flower-like morphologies.

The anti-inflammatory activity of the ZnO nanostructures was evaluated, showing a high response to protein denaturation. The sample synthesized with 20 mL of the extract showed the highest activity. The nanoparticles synthesized with 10 and 20 mL of *Sargassum natans I* alga extract demonstrated a high AA against Gram (+) *S. aureus* and moderate AA behavior against Gram (−) *P. aeruginosa,* attributed to the high surface area due to the presence of uniform nanoparticles and to the ROS species generated. Finally, the photocatalytic activity of the ZnO sample synthesized with the highest extract content and without extract was evaluated by the degradation of malachite green and methyl violet. The results showed that the nanostructures of the ZnO-50 sample had a higher degradation capacity than the flakes obtained without adding extract. In the case of malachite green, the exposure to solar radiation caused an increase in the rate of degradation. According to kinetic models, methyl violet adsorption occurred by mass transfer, while malachite green involved electron transfer. Therefore, the *Sargassum natans I* alga extract could be used to synthesize ZnO nanostructures of several well-controlled morphologies that could be used in environmental and biological applications, constituting a safe, scalable, and environmentally friendly method.

## Figures and Tables

**Table 1:** Lattice parameters and crystallite sizes of ZnO nanostructures synthesized with different *Sargassum natans I* alga extract content.

Sample	Lattice Parameters (nm)		Crystallite Size (nm)
	a	c	
ZnO-0	0.32423	0.51924	36.31
ZnO-5	0.32507	0.52069	28.46
ZnO-10	0.32520	0.52091	24.53
ZnO-20	0.32498	0.52061	21.52
ZnO-50	0.326228	0.52252	20.94

**Table 2:** Percentage of protein denaturation inhibition from all synthesized ZnO nanoparticles using *Sargassum natans I* alga extract and DFS as the positive control.

Concentration (μg/mL)		Inhibition (%)				
	Natans I	ZnO-0	ZnO-10	ZnO-20	ZnO-50	Diclofenac
100	2.92	31.39	27.52	31.47	16.32	28.35
200	6.59	48.35	42.09	49.62	30.08	40.10
300	26.98	58.64	51.87	58.79	46.97	57.65
400	36.00	72.95	70.24	75.26	67.50	71.80
500	49.09	89.07	82.70	90.01	79.68	86.21
		Concentration (μg/mL)				
IC_50_	511.13	228.10	264.78	222.78	311.59	253.76
IC_90_	839.81	514.02	553.59	503.09	555.35	525.13

**Table 3:** Percentage of protein denaturation inhibition from all synthesized ZnO nanoparticles using *Sargassum natans I* alga.

			Methyl Violet		Malachite Green	
Kinetic Model	Sample	Constant and Correlation Coefficient	Led	Solar	Led	Solar
PFO	ZnO-0	*k*_1_	0.0286	0.0127	0.0228	0.0249
		*q*_cal1_	4.0004	10.0972	14.5111	9.7051
		*R* ^2^	0.9064	0.8568	0.9617	0.8848
	ZnO-50	*k*_1_	0.0269	0.0210	0.0269	0.0306
		*q*_cal1_	3.8371	14.0896	15.9001	15.5991
		*R* ^2^	0.9268	0.9633	0.9744	0.9552
PSO	ZnO-0	*k*_2_	0.0003	0.0004	0.0015	0.0029
		*h*	0.0353	0.1490	0.6148	1.0080
		*q*_cal2_	11.1111	19.1571	20.0401	18.7970
		*R* ^2^	0.1229	0.4994	0.9652	0.9968
	ZnO-50	*k*_2_	0.0013	0.0012	0.0017	0.0031
		*h*	0.0512	0.4399	0.7487	1.5615
		*q*_cal2_	6.2972	19.5313	21.2314	22.3214
		*R* ^2^	0.9267	0.9489	0.9896	0.9971
IPD	ZnO-0	*k_id_*	0.2877	0.8857	1.4368	1.4667
		*C_i_*	−0.2981	−0.6289	1.0477	1.9028
		*R_i_*	1.1026	1.0602	0.9359	0.8844
		*R* ^2^	0.9169	0.9152	0.9688	0.9216
	ZnO-50	*k_id_*	0.2943	1.3199	1.5789	1.7721
		*C_i_*	−0.1915	0.4331	1.2347	2.9096
		*R_i_*	1.0625	0.9711	0.9294	0.8563
		*R* ^2^	0.9728	0.9903	0.9650	0.9118

**Table 4:** Model constant and correlation coefficient for Langmuir and Freundlich isotherm models for the adsorption of MV and MG onto ZnO nanostructures.

			Methyl Violet		Malachite Green	
Isotherm	Sample	Parameter	Led	Solar	Led	Solar
Langmuir	ZnO-0	*q_m_*	3.5224	16	625	−97.0874
		*k_L_*	0.3395	0.1253	0.0018	−0.0100
		*R* ^2^	0.9927	0.9567	−0.2732	0.1412
	ZnO -50	*q_m_*	3.6805	33.1126	−181.8182	714.2857
		*k_L_*	0.3047	0.0578	−0.0060	0.0019
		*R* ^2^	0.9737	0.9616	0.2660	0.3246
Freundlich	ZnO-0	*k_F_*	1.2829	2.4177	1.1418	0.8976
		*1*/*n*	0.3193	0.5582	0.9739	1.0870
		*R* ^2^	0.9451	0.924	0.9957	0.9869
	ZnO-50	*k_F_*	1.1860	2.2121	1.0254	1.3941
		*1*/*n*	0.3633	0.7321	1.0564	0.9877
		*R* ^2^	0.7944	0.9788	0.9981	0.9997

## Data Availability

The data presented in this study are available in [*Sargassum natans I* algae: An alternative for a greener approach for the synthesis of ZnO nanostructures with biological and environmental applications].

## Supplementary Materials

The following supporting information can be downloaded at: https://www.mdpi.com/article/10.3390/md21050297/s1, Figure S1: Determination of band gap obtained from the UV-Vis spectra using the Tauc equation; Figure S2: Antibacterial activity of *Sargassum natans I* alga extract by microdilution method after 3 h of contact against *S. aureus* and *P. aeruginosa*; Figure S3: Different species of *Sargassum* algae that arrive in the Caribbean: (a) *natans I* (b) *natans VIII*, and (c) *Fluitans III*; Figure S4: Typical image of the *Sargassum natans I* alga extract.
