# Supplementary material for: Sargassum natans I Algae: An Alternative for a Greener Approach for the Synthesis of ZnO Nanostructures with Biological and Environmental Applications
Source: Mar Drugs. 2023 May 13;21(5):297. doi: 10.3390/md21050297 (PMC10224490; doi:10.3390/md21050297)
Supplement: Supplementary file 1 [file marinedrugs-21-00297-s001.zip › marinedrugs-2362272-supplementary.pdf]

Supplementary Materials:

Sargassum natans I algae: An Alternative for a Greener Approach for the Synthesis of ZnO Nanostructures with Biological and Environmental Applications

Jose Luis López-Miranda [1], Fabian Mares-Briones [1], Gustavo A. Molina [1], M. A. González-Reyna [1], Isaac Velázquez-Hernández [1], Beatriz Liliana España-Sánchez [2], Rodolfo Silva [3], Rodrigo Esparza [1] and Miriam Estévez [1,*]

[1] Centro de Física Aplicada y Tecnología Avanzada, Universidad Nacional Autónoma de México, Boulevard Juriquilla 3001, Querétaro 76230, Mexico; lopezfim@gmail.com (J.L.L.-M.); fabianmares@gmail.com (F.M.-B.); gamol@fata.unam.mx (G.A.M.); marlengonzalez@fata.unam.mx (M.A.G.-R.); isaacvh_qi@hotmail.com (I.V.-H.); resparza@fata.unam.mx (R.E.)

[2] CONACYT_Centro de Investigación y Desarrollo Tecnológico en Electroquímica SC, Parque Tecnológico Querétaro s/n Sanfandila, Pedro Escobedo 76703, Mexico; lespana@cideteq.mx

[3] Instituto de Ingeniería, Universidad Nacional Autónoma de México, Edificio 17, Ciudad Universitaria, Coyoacán, Mexico City 04510, Mexico; rsilvac@iingen.unam.mx

* Correspondence: miries@fata.unam.mx

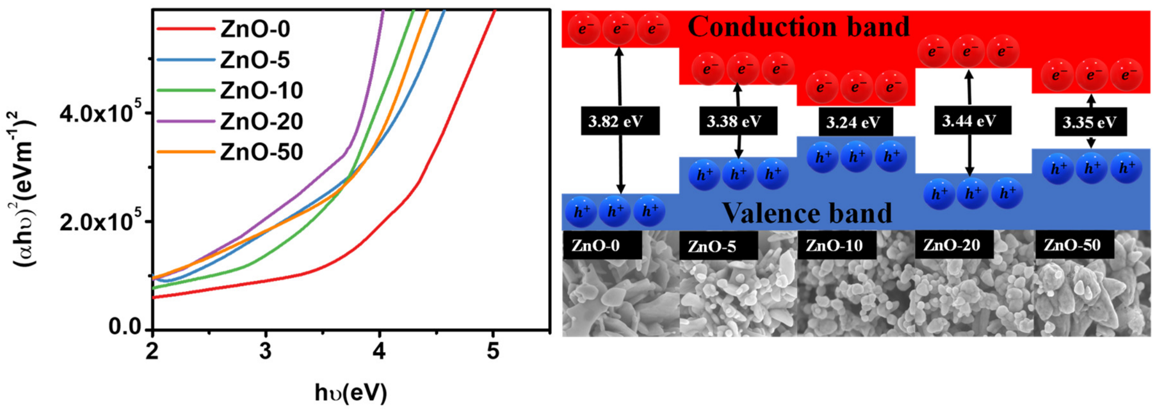

Figure S1. Determination of band gap obtained from the UV-Vis spectra using the Tauc equation.

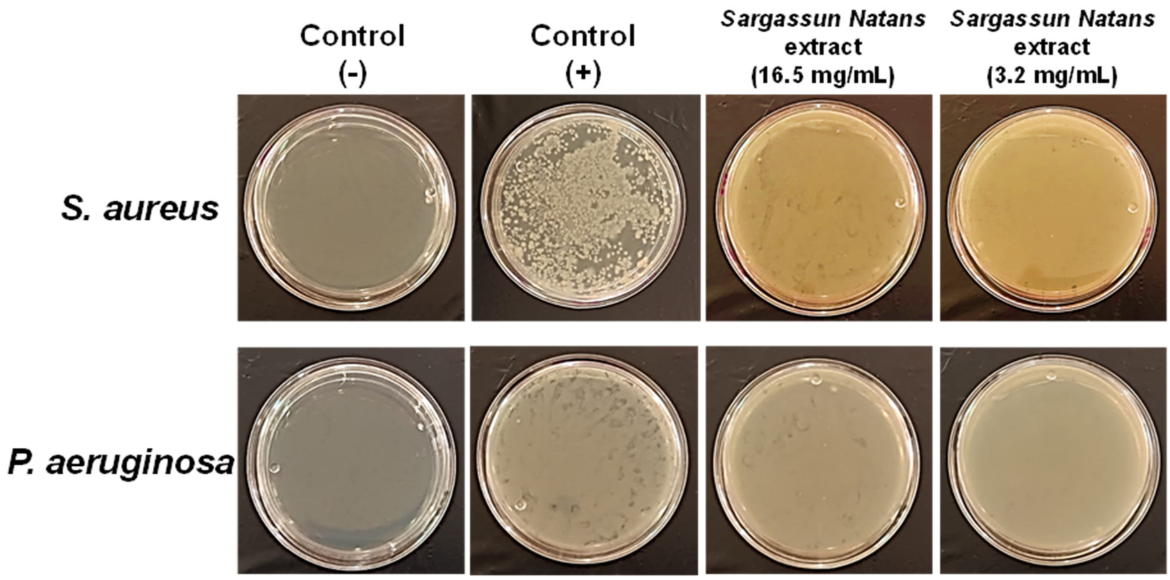

Figure S2. Antibacterial activity of *Sargassum natans I* alga extract by microdilution method after 3 h of contact against *S. aureus* and *P. aeruginosa*.

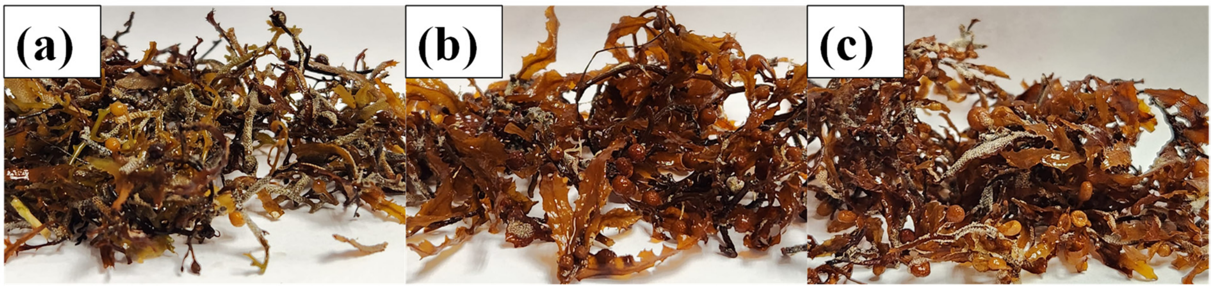

Figure S3. Different species of *Sargassum* algae that arrive in the Caribbean: (a) *natans I*, (b) *natans VIII*, and (c) *Fluitans III.*

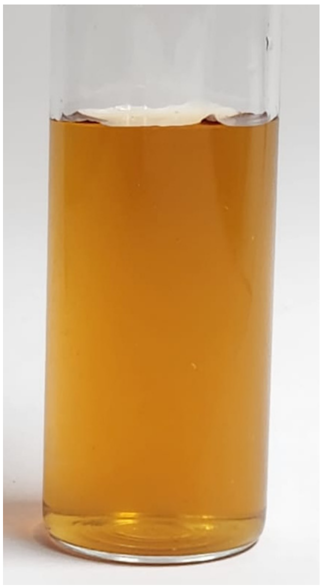

Figure S4. Typical image of the *Sargassum natans I* alga extract.
